# Relationship of stunting with water, sanitation, and hygiene (WASH) practices among children under the age of five: a cross-sectional study in Southern Punjab, Pakistan

**DOI:** 10.1186/s12889-023-17135-z

**Published:** 2023-11-03

**Authors:** Munazza Batool, Javeria Saleem, Rubeena Zakar, Muhammad Salman Butt, Sanaullah Iqbal, Shahroz Haider, Florian Fischer

**Affiliations:** 1 Department of Public Health, University of the Punjab, Lahore, Pakistan; 2 Department of Food Science and Human Nutrition, University of Veterinary and Animal Sciences, Lahore, Pakistan; 3 Bakhtawar Amin Medical and Dental College Multan, Multan, Pakistan; 4 Institute of Public Health, Charité – Universitätsmedizin Berlin, Berlin, Germany

**Keywords:** Underweight, Undernutrition, Impaired growth, Developmental delay

## Abstract

**Background:**

Reasons for undernutrition are food insufficiency, impaired child care, limited access to healthcare, and maternal lack of health literacy. In addition, there are several environmental factors, such as drinking water quality, poor sanitation, and hygienic practices that can lead to poor nutritional status in children. The present study aimed to compare household-associated risk factors, including water, sanitation, and hygiene (WASH) practices of mothers, with children’s stunting under the age of five.

**Methods:**

A face-to-face cross-sectional survey was conducted with mothers of children under the age of five in the Dera Ghazi Khan district in southern Punjab, Pakistan. World Health Organization criteria for stunting were used to identify the participants. A sociodemographic questionnaire was used to collect information from consented parents/guardians on children’s age, feeding, and WASH practices. Pearson’s chi-square, simple regression, and hierarchical regression models were applied for data analysis.

**Results:**

A total of 204 mothers of children of both genders participated in this study. The children’s mean (SD) age was 15.67 (± 10.2) months, their weight was 5.44 (± 1.45) kg, their height was 67.69 (± 10.05), and their mid-upper arm circumference was 9.75 (± 1.30) cm. Children’s stunting was mild (z-score > -2) at 17.6%, moderate (z-score − 2 to -3) at 16.7%, and severe (z-score < -3) at 65.7% of participants. A simple regression model showed a strong association, r^2^ = 0.062, p = 0.013, with age as the most significant sociodemographic factor. The hierarchical regression model showed a combined value of r^2^ = 0.0128, p = 0.027, with hand pump and tank water as the significant source of drinking water that was related to stunting among children.

**Conclusion:**

Stunting can be associated with several risk factors, including WASH parameters. This study concluded that children aged under five years are susceptible to stunting in southern Punjab, Pakistan. The contamination of hand pumps and tank water resources was found to be the major contributing factor to stunting.

## Background

Undernutrition is one of the major global public health issues causing premature morbidities and mortalities among children under five years of age, especially in developing countries [1]. Globally, in 2020, 149.2 million children aged less than five years were stunted, 45.4 million had a prevalence of wasting, and 38.9 million were overweight [2]. Evidence shows that nearly 45% of deaths among these children are linked to undernutrition, which makes them vulnerable to common infections, delays recovery, and even death [2, 3]. Hunger and undernutrition are estimated to be the primary cause of half of the mortality among children around the globe. The majority of these cases arise in low- to middle-income countries of Asia and Africa (55% and 39%, respectively) [4]. The prevalence of malnutrition is alarming in Pakistan, with an estimated 65.2 deaths per thousand live births accounting for three million deaths annually amomg children under five years of age [5]. The prevalence of stunting was estimated to be 45%, wasting 10.5%, and being underweight at 31.6% in Pakistan according to the UNICEF and World Health Organization (WHO) joint report 2016 [2].

Literature highlights that one of the major contributors to malnutrition among children under five years of age is poor sanitary and hygienic conditions. Poor sanitary and hygienic conditions in houses result in chronic exposure to environmental pathogens, which causes changes in gut microbiota morphology and function which can lead to stunting among children [4, 6]. Stringent evidence between environmental factors and chronic inflammatory syndrome of the gut is present, and this condition is known as pediatric environmental enteropathy [6]. These diseases further affects the development and growth of children and disrupts their gut microbiota [7]. Diarrhea is considered to be a major contributing factor to malnutrition, which is caused by pathogens of gut microbiota. Subclinical variations in the gut microbiome can cause stunting even though there are no obvious infections, such as diarrhea [8, 9].

Extreme poverty and lack of sanitation facilities can easily cause enteric infections among children aged under two years [10]. Such enteric infections can lead to stunting and wasting as a result of malabsorption and barrier dysfunction of the intestine [11]. Therefore, the height of a child at their second birthday is considered a predictor of cognitive development and overall health [12]. Further evidence reveals that sociodemographic, environmental, cultural, and economic nutritional factors are important contributors to poor health conditions among children under five years of age [13]. Literature indicates that undernutrition can indirectly be caused by food insufficiency, impaired child care, limited access to healthcare, maternal illiteracy, and environmental factors, including the access to and quality of drinking water, sanitation, and hygiene (WASH) practices [14]. Repeated ingestion of fecal bacteria through contaminated water and poor hygienic practices can cause overloading of the small intestine and malabsorption, mucosal leakage, impaired villi functions, and gut cell inflammation as a result of environmental enteropathy [15].

Previous research has rendered the relationship between stunting and WASH practices in low- to middle-income countries. The phenomenon of environmental enteropathy has also been addressed in some studies, but limited evidence is available from Pakistan. Therefore, the present study aimed to compare household environmental-associated risk factors, including water, sanitation, and hygiene (WASH) practices of mothers, with children stunting under the age of five in southern Punjab, Pakistan.

## Methods

### Study design and setting

This cross-sectional study measures the water, sanitation, and hygiene practices of children under five and mothers living in the southern Punjab of Pakistan. The data were collected from March 2020 to June 2021.This region comprises three divisions and 11 districts. The total area of South Punjab is 99,572 km, which is 48.5% of the entire Punjab region. The approximate population of South Punjab is 34.7 million. An estimated 31% of the population of southern Punjab lives below the national poverty line while 55% population live below half of Pakistan’s median per capita income. The region has the highest rates of poverty, stunting and wasting as compared to rest of the Punjab [16]. Fifty-two functional outpatient therapeutic program centers (OTPs) in the Dera Ghazi Khan district are administered by the “National Program for Family Planning and Primary Health Care”. The OTP center of the teaching hospital Dera Ghazi Khan was selected for data collection. The selected area is underdeveloped, with substandard housing due to poverty and illiteracy, overcrowded poor socioeconomic conditions, malnutrition, and unhygienic living conditions [17]. The data were collected by the consultant pediatrician with the help of a lady health worker.

### Study population and sampling

**Fig. 1:**
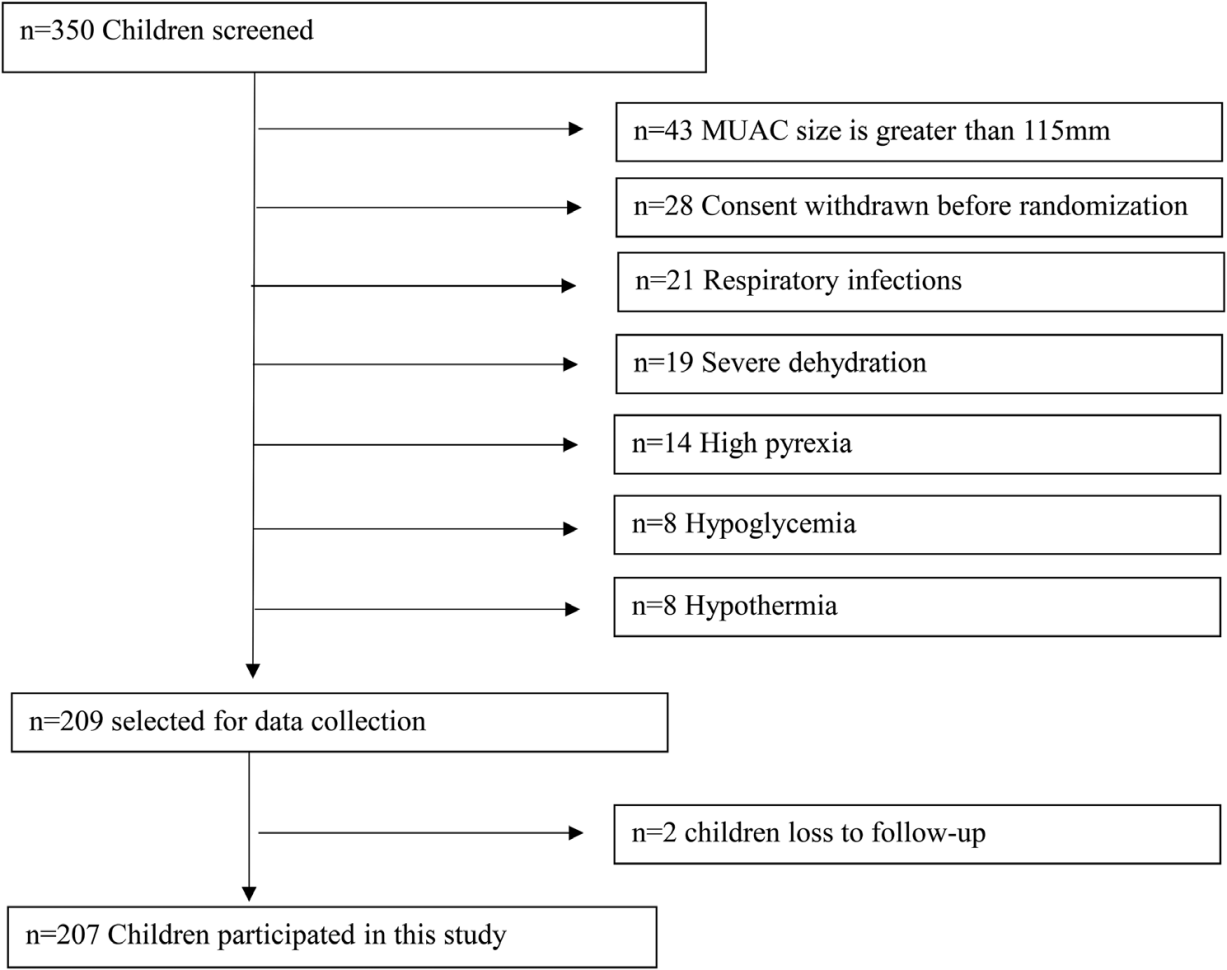
Flow chart of sampling procedure

Stunted children aged 6 to 59 months living in the Dera Ghazi Khan district were selected using predefined inclusion and exclusion criteria for this study. WHO criteria of mid-upper arm circumference (MUAC) < 115 mm or weight-for-height z-score ≤ -3 or grade 1 to 2 bilateral edemas, clinically well children, alert, and good appetite were used for the identified study population [18]. Exclusion criteria of severe dehydration, pitting edema, hypothermia anorexia, hypothermia, high pyrexia, acute lower respiratory infection, or hypoglycemia were used in this study. A total of 350 children were screened for baseline assessment, and 209 children were found eligible to participate in this study, as shown in the flow chart in Fig. 1.

### Sample size determination

The sample size was calculated by using the WHO sample size determination in health sciences software version 2.0 [19]. The sample size estimation was performed for a population-specified relative precision. A prevalence of 45% for stunting was used to anticipate the population proportion (P) of 0.45 with a relative precision of (ε) 0.15 with a confidence interval (1-α) of 95% to determine the sample size. A sample size of 209 was calculated, and data collected from 204 participants in this study accounted for a non-response rate of < 3%.

### Data collection procedures and measurement of variables

A survey questionnaire was used to collect information from consented parents/guardians on children’s age, feeding and WASH practices, presence of any comorbidity two weeks before participation in this study, and availability of the pets in the house. Water quality biochemical analysis for the nearest available water resources was also performed. Researchers’ subjective assessment was used to rate the general appearance of the mother and children’s hands and clothes.

The anthropometric variables of height, weight, and MUAC were measured by the lady health workers. A specific color-labelled tape was used to measure the mid-upper arm circumference of the child, and the measurements were rounded to the nearest 0.1 cm. The 2-in-1 function of the Seca 874 U electronic scale was used to measure the weight of infants aged less than two years with the help of their mother/guardian. The weight of a child aged greater than two years was measured using the Seca 336 baby scale. Weight was measured with essential clothing and shoes removed and rounded to the nearest 100 g. The height of the children aged less than two years was measured using the Seca 417 length measure scale, and the Seca 213 height measure scale was used for older children. The measurements were rounded to the nearest 0.1 cm. The z-score was calculated by using the height for age (stunting) formula by using WHO Anthro v3.2.2 software [20].

One nurse was instructed to obtain consent for the collection of blood and stool samples and send them to the laboratory. Drinking water was sent to a water testing laboratory for a subset of 118 drinking water sources for chemical analysis and coliform. The turbidity of the samples was analysed using the nephelometric method with a turbidity meter (Lamotte-2008, USA).

Mother’s knowledge was also measured for infant and young child feeding practices along with their hygienic habits of hand washing before eating food and after using the toilet. A brief medical history of the child was also measured to estimate the visits to the hospital or any health service center to seek medical assistance. BCG scarring was observed to evaluate the child’s immunization status.

### Data processing

All the duly filled forms were collected daily and were manually verified by the principal investigator to rectify the data errors if applicable. Incomplete forms were completed by consulting the mother again. Verified data were entered into the Statistical Package for the Social Sciences (SPSS) version 23 sheet for further processing [21]. Data were entered by the principal investigator and cross-checked by the coauthors. In case of any error or incorrect entry, the data were verified again before statistical data analysis. A backup depository was developed to store the data and prevent any data loss. Prior to analyzing the data, the data were cleaned and coded.

### Data analysis

A descriptive statistical analysis was performed on the sociodemographic variables of the children. A cross-tabulation Pearson’s chi-square analysis was performed to measure the significant relationship (p < 0.05) between the WASH predictors and stunting. A simple linear and hierarchical regression model combination was applied to evaluate the determinants of stunting and the effects of WASH predictors on stunting. Simple regression models were applied to measure the association between the WASH predictors by considering the stunting z-score as the outcome variable. This model determines the association strength (r^2^) between the stunting and the WASH predictors. Hierarchical regression models were applied to strongly associated WASH predictors by considering the sociodemographic variables of age, sex, ethnicity, and area of residence as confounding factors.

### Ethical considerations

The Advanced Studies Board (ASRB) (Ref # D-150/ACAD) of the University of the Punjab, Lahore Pakistan approved this research project. Ethics approval was obtained from the Institutional Review Board (IRB) (ref # D/193/DFEMS). The ethical considerations of the Helsinki Declaration were also observed to recruit the patients and data collection [22].

## Results

A total of 204 children of both genders participated in this study. The children’s mean (SD) age was 15.67 (± 10.2) months, weight was 5.44 (± 1.45) kg, height was 67.69 (± 10.05), and MUAC was 9.75 (± 1.30) cm (Table 1). Children’s stunting was mild (z-score > -2) at 17.6%, moderate (z-score − 2 to -3) at 16.7%, and severe (z-score < -3) at 65.7% of participants (Table 2). A cross-tabulation using the Pearson’s chi-square test showed a non-significant (p > 0.05) relationship between the sociodemographic variables and the WASH predictors with stunting (Table 3).

**Table 1:** Socio-demographic characteristics of the children under five (n = 204)

Variables	Groups	n (%)
Gender	Boys	108 (63.7)
	Girls	96 (47.1)
Age 15.67 ± 10.2 months	Up to 12 months	104 (51.0)
	13–24 months	71 (34.8)
	25–36 months	21(10.3)
	37–48 months	5(2.5)
	49–60 months	3(1.5)
Ethnicity	Baloch	68 (33.3)
	Migrant	13 (6.4)
	Native	123(60.3)
Resident	Rural	130 (63.7)
	Semirural	13 (6.4)
	Urban	61(29.9)
Weight of child 5.44 ± 1.45 kg	2.4–5.0 kg	83 (40.7)
	5.1–7.5 kg	108 (52.9)
	7.6–10.0 kg	13 (6.4)
Length 67.69 ± 10.05 cm	45–65 cm	94 (46.1)
	66–85 cm	100 (49.0)
	86–105 cm	10 (4.9)
Mid-upper arm circumference 9.75 ± 1.30 cm	6.5–8.0	36 (17.6)
	8.1–10.0	88 (43.1)
	10.1–12.0	80 (39.2)

**Table 2:** Relationship between the stunting and WASH variables

		Severity of stunting			Total	Pearson’s Chi-square
		Mild (<-2 z-score)	Moderate (≤-2 & ≥-3 z-score)	Severe (<-3 z-score)		p-value
Sex	Male	24 (11.8%)	16 (7.8%)	68 (33.3%)	108 (52.9%)	0.178
	Female	12 (5.9%)	18 (8.8%)	66 (32.4%)	96 (47.1%)	
Age	< 12 months	13 (6.4%)	15 (7.4%)	76 (37.5%)	104 (51.0%)	0.061
	≥ 12 months	23 (11.3%)	19 (9.3%)	58 (28.4%)	100 (49%)	
Water quality	Poor quality drinking water	15 (7.4%)	13 (6.4%)	63 (30.9%)	91 (44.6%)	0.607
	Improved quality drinking water	21 (10.3%)	21 (10.3%)	71 (34.8%)	113 (55.4%)	
Pets in the house	Yes	17 (8.3%)	13 (6.4%)	52 (25.5%)	82 (40.2%)	0.637
	No	19 (9.3%)	21 (10.3%)	82 (40.2%)	122 (59.8%)	
Hygienic condition of child	Clean	8 (3.9%)	6 (2.9%)	38 (18.6)	52 (25.5%)	0.390
	Unclean	28 (13.7%)	28 (13.7%)	96 (47.1%)	152 (74.5%)	
Prevalence of pre-existing morbidities diarrhoea	Often	32 (15.7%)	31 (15.2%)	125 (61.3%)	188 (92.2%)	0.670
	No	4 (2.0%)	3 (1.5%)	9 (4.4%)	16 (7.8%)	
Hygienic condition of mother	Clean	8 (3.9%)	6 (2.9%)	38 (18.9%)	52 (25.5%)	0.390
	Unclean	28 (13.7%)	28 (13.7%)	96 (47.1%)	152 (74.5%)	
Toilet in the house	Yes	32 (15.7%)	31 (15.2%)	117 (54.4%)	180 (88.2%)	0.816
	No	4 (11.1%)	3 (1.5%)	17 (8.3%)	134 (65.7%)	
Total		36 (17.6%)	34 (16.7%)	134 (65.7%)	204 (100%)	

**Table 3:** Relationship between the severity of stunting and water source

		Severity of stunting			Total	Pearson’s Chi-square
		Mild (<-2 z-score)	Moderate (≤ -2 & ≥ -3 z-score)	Severe (<-3 z-score)		p-value
Ponds	Yes	3 (1.5%)	1 (0.5%)	11 (5.4%)	15 (7.4%)	0.558
	No	33 (16.2%)	33 (16.2%)	123 (60.3%)	189 (92.6%)	
Hand pump	Yes	25 (12.3%)	24 (11.8%)	93 (45.6%)	142 (69.6%)	0.991
	No	11 (5.4%)	10 (4.9%)	41 (20.1%)	62 (30.4%)	
Filteration plan	Yes	6 (2.9%)	8 (3.9%)	26 (12.7%)	40 (19.6%)	0.766
	No	30 (14.7%)	26 (12.7%)	108 (52.9%)	164 (80.4%)	
Water supply	Yes	2 (1.0%)	5 (2.5%)	21 (10.3%)	28 (13.7%)	0.289
	No	34 (16.7%)	29 (14.2%)	113 (55.4%)	176 (86.3%)	
Tank water	Yes	7 (3.4%)	10 (4.9%)	42 (20.6%)	59 (28.9%)	0.375
	No	29 (14.2%)	24 (11.8%)	92 (45.1%)	145 (71.1%)	

Further, a hierarchical regression analysis was applied in two models. In model 1, age, sex, ethnicity, and area of residence were added. Model 1 showed a strong association (r^2^ = 0.062, p = 0.013), with age as the onlysignificant predictor (B = 0.201; 95% CI: 0.07–0.41; p < 0.001). A combined model (model 2) was applied with the sources of water and stunting as an outcome by considering the sociodemographic variables. This model showed a combined value of r^2^ = 0.128, p = 0.027 with hand pump (B = 0.356; 95% CI: 0.23–1.37; p < 0.001) tank water (B = 0.37; 95% CI: 0.27–1.43; p < 0.001) and age (B = 0.211; 95% C: 0.09–0.42; p < 0.001) as the significant predictors of stunting among children, as shown in Table 4.

**Table 4:** Simple regression analysis of WASH determinants with stunting z-score

Model		Beta coefficients	Sig. value	95% Confidence interval for B		R^2^ for model	p-value
				Lower bound	Upper bound		
Model 1	Ethnicity	0.083	0.238	-0.061	0.246	0.062	0.013*
	Age of the child	0.201	0.004*	0.077	0.408		
	Sex	-0.085	0.226	-0.462	0.110		
	Resident	-0.118	0.095	-0.293	0.024		
Model 2	Ethnicity	0.123	0.088	-0.021	0.295	0.128	0.027*
	Age of the child	0.211	0.003*	0.089	0.417		
	Sex	-0.069	0.318	-0.426	0.139		
	Resident	-0.097	0.225	-0.291	0.069		
	Water source - Water supply	0.132	0.074	-0.039	0.836		
	Water quality	0.129	0.065	-0.017	0.557		
	Water source - Ponds	0.003	0.966	-0.541	0.565		
	Water source - Hand pump	0.356	0.006*	0.234	1.371		
	Water source - Filtration plant	-0.076	0.352	-0.616	0.220		
	Water Source - Tank water	0.372	0.004*	0.276	1.428		

* indicates significant results (p < 0.05)

## Discussion

This study evaluated the key stunting determinants among children under five years of age, including WASH predictors and sociodemographic variables, in southern Punjab, Pakistan. The WASH key determinants of water source, water quality, sanitation, pets in the house, and hygiene practices of mothers and children were measured and compared with the severity of stunting. The sociodemographic variables of age, sex, and area of residence were measured and correlated with stunting severity among children aged under five years. Severe stunting was observed among children aged < 12 months. The hierarchical regression models showed that among all these sociodemographic factors, age had a significant (r^2^ = 0.062, p = 0.013) relationship with stunting. This finding highlights the importance of the combined effects of environmental enteropathy, which can collectively be a possible contributing factor for stunting in this age group.

The gender of the child showed a non-significant relationship in this study for stunting. Previous studies had mixed findings on this sociodemographic factor, as some studies showed similar non-significant results, whereas some considered gender to be an important contributing factor in stunting among children under five years. In one study conducted in Pakistan, gender was found to be a significant predictor of growth and retardartion among children [23]. Likewise, a study from India, reported sex differences to be a significant factor of nutritional status, with girls found to be more prone to stunting [24]. Contrary to these findings, some studies conducted in Ethiopia found no significant difference between the sex of the child and the stunting among children under five years of age [25]. Gender differences in malnutrition status among children are well-documented in Pakistan with some studies reporting females to be more vulnerable for malnutrition [26] while the other studies reported the higher likihood of male children to be underweight as compared to female children [27]. No gender differences among malnutritional status of children found in the present study could be due the fact that data for the present study were collected during COVID-19 pandemic period, which subjected everyone to a vulnerable state despite of their wealth index and gender. One study conducted in Pakistan also revelaed that reduced wealth disparities causes decrease in gender disparities in nutritional status among children [28].

The findings of this study were similar to those of previous studies conducted in other underprivileged regions. Inadequate nutritional resources, lack of clean drinking water, and poor sanitation conditions among these underprivileged areas are common and can be considered major contributing factors for stunting among children under five years. A similar study conducted in rural Ethiopia found age to be a significant factor in stunting when children remained exposed to an inadequate environment and nutrition [29]. The hygiene condition of the children determines the child’s health, growth, and development. Studies have suggested that WASH is not limited to toilet facilities, hand washing practices, and water purification but should also be considered from hygiene-related physical and environmental perspectives [30]. The results of the present study showed that the physical sources of drinking water had a strong significant relationship with stunting, with the hand pump and tank water sources found to be unsafe for drinking that can contribute to the stunting occurrence. The contamination of these sources is common and can prove to be a potential source of dysentery and gastroenteritis among children. Studies highlight that contaminated water carries different pathogens that can cause different diarrheal diseases including campylobacteriosis, giardiasis, gastroenteritis, amoebiasis and cholera [31].

This study presented an outline of the sociodemographic and WASH predictors in southern Punjab, Pakistan, and tried to develop a context-specific association from a regional perspective. The study is very unique in its own kind, because as per the resaerchers’ knowledge there is very limited evidence available regarding WASH practices and stunting among children from southern Punjab, Pakistan. The study had some limitations to account for in future research and better evaluation of these determinants. Firstly, the data for the present study were collected during the COVID-19 pandemic period in Pakistan. It is well understood fact that the socioeconomic situation, the administrative actions of the health system and municipality of the country were badly affected by pandemic. The data collected during the pandemic period could have aggrevated the effects of sociodemographic factors that may not be generalizeable in normal conditions. Another limitation was that the study was confined to the southern Punjab region, and future research from other underprivileged regions of Pakistan for further comparison is recommended. Furthermore, this study did not include the nutritional assessment of the children, effects of microbial ingestion, and effects of prebiotics (galactooligosaccharides).

## Conclusion

Stunting can be associated with several risk factors, including WASH parameters. This study concluded that children aged under five years are susceptible to stunting in southern Punjab, Pakistan. The contamination of hand pumps and tank water resources was found to be the major contributing factor to stunting. Therefore, there is a need to ensure the availability of safe and clean drinking water in underprivileged areas, and the source of water should be physically and biochemically monitored to promote the health and well-being of the population.

## Data Availability

The dataset used during the current study is available from the corresponding author on reasonable request.

## Abbreviations

MUAC: Mid-upper arm circumference
OTP: Outpatient therapeutic program
SD: Standard deviation
WASH: Water, sanitation, and hygiene
WHO: World Health Organization
